# Estimation of Energy Losses in Nanocrystalline FINEMET Alloys Working at High Frequency

**DOI:** 10.3390/ma14247745

**Published:** 2021-12-15

**Authors:** Lucian-Gabriel Petrescu, Maria-Catalina Petrescu, Emil Cazacu, Catalin-Daniel Constantinescu

**Affiliations:** 1 Department of Electrical Engineering, Faculty of Electrical Engineering, University “POLITEHNICA” of Bucharest, 313 Splaiul Independentei, RO-060042 Bucharest, Romania; catalina.petrescu@upb.ro (M.-C.P.); emil.cazacu@upb.ro (E.C.); 2 Laboratoire LP3/UMR 7341, CNRS, Aix-Marseille Université, F-13009 Marseille, France

**Keywords:** FINEMET, soft magnetic alloy, Steinmetz losses estimation, nanocrystalline ribbon, planar transformers, high frequency applications

## Abstract

Soft magnetic materials are at the core of electromagnetic devices. Planar transformers are essential pieces of equipment working at high frequency. Usually, their magnetic core is made of various types of ferrites or iron-based alloys. An upcoming alternative might be the replacement the ferrites with FINEMET-type alloys, of nominal composition of Fe_73.5_Si_13.5_B_9_Cu_3_Nb_1_ (at. %). FINEMET is a nanocrystalline material exhibiting excellent magnetic properties at high frequencies, a soft magnetic alloy that has been in the focus of interest in the last years thanks to its high saturation magnetization, high permeability, and low core loss. Here, we present and discuss the measured and modelled properties of this material. Owing to the limits of the experimental set-up, an estimate of the total magnetic losses within this magnetic material is made, for values greater than the measurement limits of the magnetic flux density and frequency, with reasonable results for potential applications of FINMET-type alloys and thin films in high frequency planar transformer cores.

## 1. Introduction

Soft magnetic materials are an essential part of electromagnetic devices, in bulk or as thin films [1,2,3,4]. Their magnetic core must withstand easy magnetization and exhibit low energy losses, the most common demands for any soft magnetic materials [5,6]. Nowadays, devices that work at high frequencies, e.g., such as thin film and/or planar transformers, also require high resistivity of the magnetic core to reduce losses [7,8]. Current technology in high frequency switching planar transformers involves the use of soft ferrites, for the core, with various chemical composition [9,10,11]. This class of ceramic materials fulfil the main requirements, such as high saturation flux density, high permeability (even for kHz frequencies), and low core losses. In addition, the ferrite cores have a typical efficiency of up to 97–99%, which offers a great heat dissipation [12].

Magnetic materials capable of operating at higher operating frequencies have the potential to greatly reduce the size of megawatt level power electronics. In the last decades, a new class of materials became more common for applications operating at high frequency: magnetic nanocrystalline compounds [13]. These are formed by an assembly of regions of coherent crystal structure (grains), with an average grain diameter of 1 to 50 nm. Exhibiting a magnetic order, they may also be embedded in a magnetic or non-magnetic matrix. Ribbons of nanocrystalline alloys are made by rapid solidification techniques, deposition, and solid state reactions, where the initial material can be in the amorphous state and then crystallize. The composition of the alloy, its crystalline structure, the microstructure, and the morphology will thus determine the magnetic properties of the material.

The name FINEMET^®^ is a commercial brand that has high saturation flux density and high permeability, and that exhibits stable temperature characteristics [14,15,16,17]. The alloy is a met-glass-like, nanocrystalline soft magnetic material developed in Japan by Hitachi Corporation, which can hold up to 2 kG of magnetic flux intensity. It is usually made into a crystalized alloy ribbon, with a main chemical composition of Fe. Starting from conventional Fe-Si-B systems, and by adding minor quantities of Cu and Nb, the Fe-Si-B-Nb-Cu type alloy, i.e., FINEMET [18,19], can be achieved. Its phase characteristics are far better than conventional core materials, such as Ni- and Co-based amorphous metals and/or alloys. Such an alloy can be produced by precipitation of the nanocrystalline α-Fe dispersed in an amorphous matrix, with saturation magnetic flux densities over 1.2 T, relative permeability higher than 1000 at frequencies of kHz, a low coercive field, and very low magnetic losses [20]. Owing to its low magnetostriction effects, it is less affected by mechanical stress and aging, and thus suitable for high frequency applications.

Typically, this nanocrystalline alloy is made of grains that are highly uniform (about 10 nm in size), prepared by single roll melt spinning technique [17,21]. Using this procedure, the alloys are rapidly cooled from the molten state to the non-crystalline (amorphous) state, thus crystallization is prevented from occurring. The resulting amorphous ribbons reach a thickness of up to 20 µm and a width that can vary, depending on the application for which they are prepared, from a few millimetres to centimetres [22]. For obtaining a nanocrystalline material, the velocity of the cooling roller is reduced.

Here, we present and discuss results on a selection of experimental determinations, for a maximum of 300 mT and 900 kHz, for a FINEMET ribbon. The experimental set-up used prevents the readings of magnetic characteristics at high magnetic flux density and high frequencies. Owing to such aspects, it is not possible to have the specific losses determined at magnetic flux densities over 300 mT. The novelty of our approach, presented in this work, is to use the Steinmetz loss modelling procedure [5] in determining a series of fitting relationships for specific parameters. In this way, we are able to estimate and discuss the total magnetic energy losses in this material and for magnetic flux densities up to 1 T, in ribbons (bulk) and for thin films.

## 2. Materials and Methods

**Figure 1:**
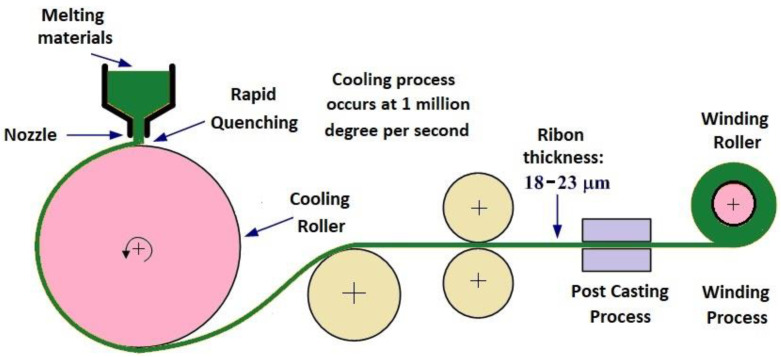
Rapid quenching method for obtaining amorphous/nanocrystalline ribbon materials.

FINEMET-D is a commercial nanocrystalline material (Fe_73.5_Si_13.5_B_9_Nb_3_Cu_1_), which suffers a mechanical treatment to reduce its thickness. Amorphous and nanocrystalline ribbons are obtained by rapid quenching and the main difference between these materials is imposed by the velocity of the cooling roller (see Figure 1). Finally, different techniques are used to obtain the ribbons, with a thickness of approximately 18–23 µm [23].

**Figure 2:**
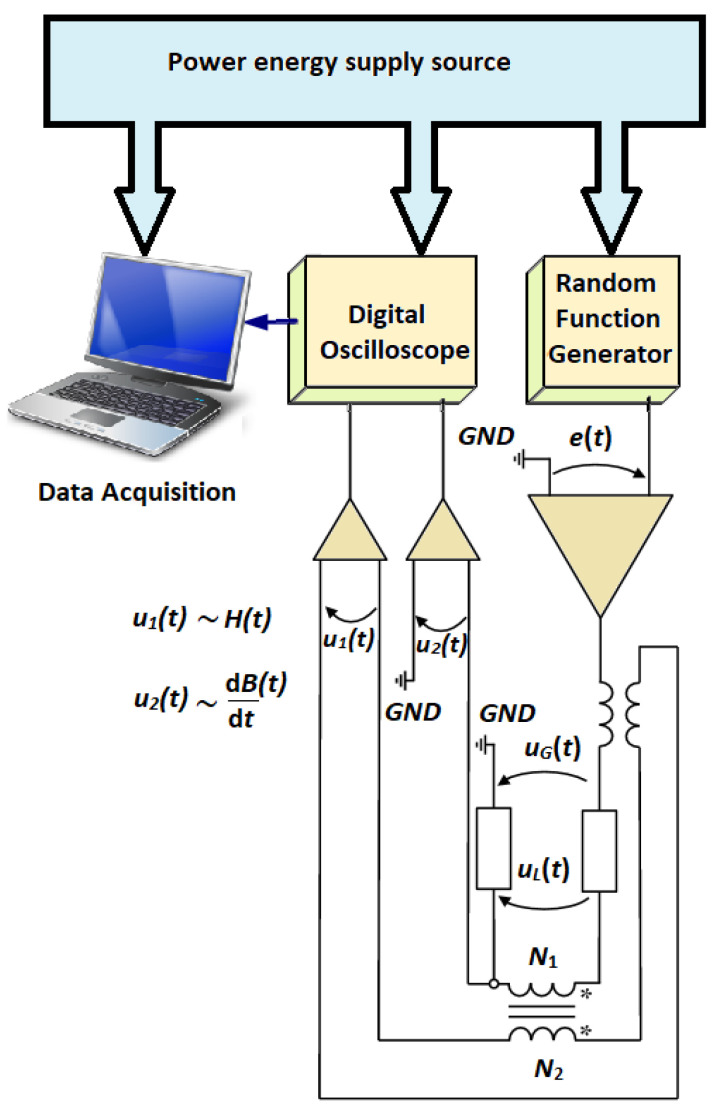
Experimental setup for measuring the magnetic properties of the FINEMET ribbon placed as the transformer core.

The experiment set-up presented in Figure 2 was developed at the INRIM Laboratories in Torino, Italy. Two voltage signals *u*_1_(t) and *u*_2_(t) are acquired from the magnetic core. The first one is proportional with the magnetic field strength *H*, while the second one is with the magnetic flux density *B* [24]. *N*_1_ and *N*_2_ represent the number of turns of the two windings that build the transformer having as core the FINEMET band.

The voltage provided by the random function generator is denoted by *e*(*t*). Using some amplifiers, all data are converted to magnetic quantity, thus the hysteresis loops are obtained.

With respect to the frequency of the signal, and thus the effective value of the supply voltage, it was necessary to make an adjustment, i.e., to determine the number of coil turns. The experimental set-up has a technical limitation, and one can manage to obtain the hysteresis loops at frequency up to 200 kHz only and for low magnetic flux densities (lower than 300 mT). However, at lower frequencies (i.e., up to 50 kHz), the saturation value of the magnetic flux density can be obtained.

**Figure 3:**
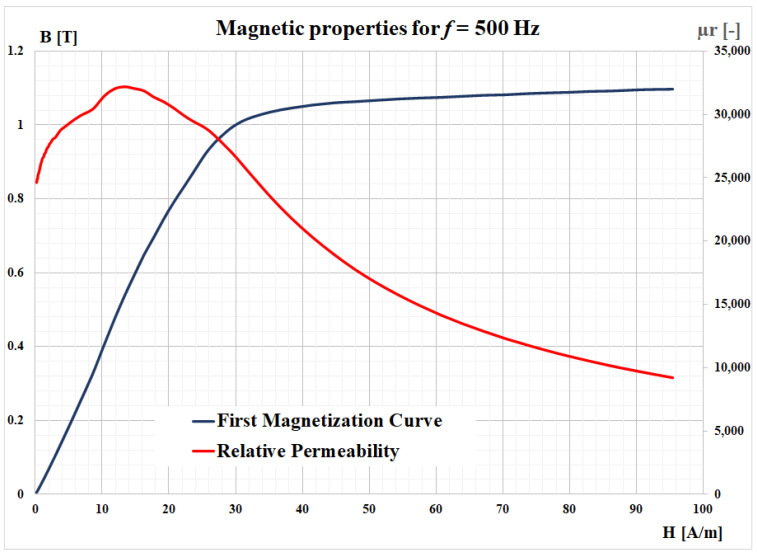
First magnetization curve and the relative permeability measured for the FINEMET ribbon for 500 Hz.

**Figure 4:**
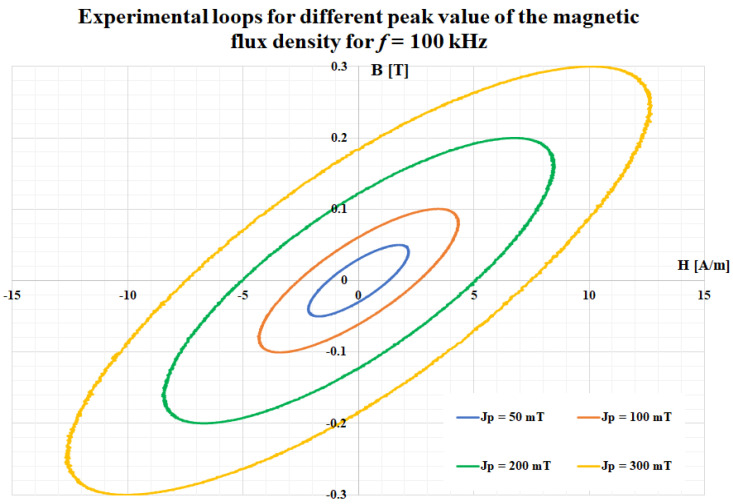
Comparison between experimental loops for different values of the magnetic flux density obtained at 100 kHz.

**Figure 5:**
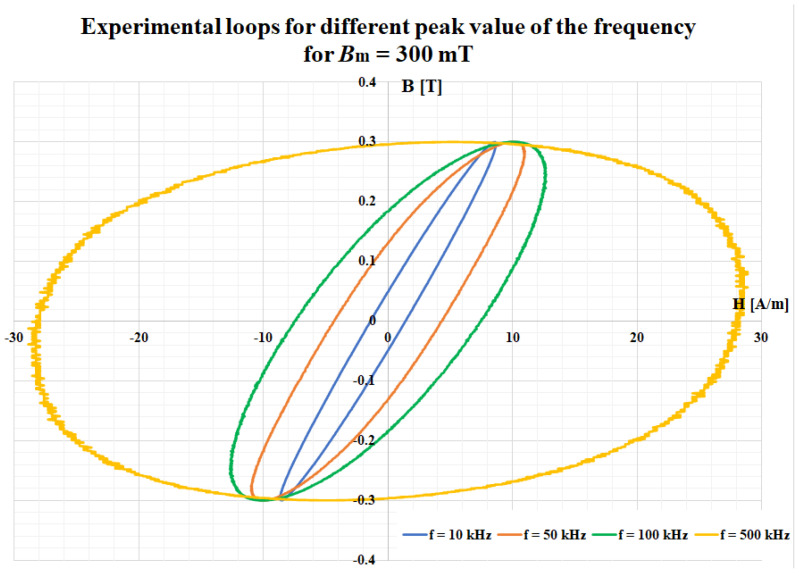
Comparison between experimental loops for different values of the frequency obtained at 300 mT.

The major magnetic properties of the ribbon (first magnetization curve and the relative permeability), obtained for an operating frequency of 500 Hz, are depicted in Figure 3. A complete characterization of this material was presented in a previously published study [24]. Figure 4 presents a comparison between the experimental hysteresis loops obtained for the FINEMET ribbon at 100 kHz and different values of the magnetic flux density. In Figure 5, a comparison is shown for the same magnetic flux density (300 mT), but for different values of the frequency.

## 3. Steinmetz Losses Estimation

Steinmetz was one of first scientists that developed a model to estimate the magnetic losses in electrical steels [25]. In the following decades, this model was further modified and improved in order to consider the evolution of soft magnetic materials [26,27,28]. Thus, the initial Steinmetz relation (1) indicated the magnetic losses’ proportionality with the hysteresis energy losses and of the value of the magnetic polarization:

(1) $$W_{\mathrm{tot}}=k_{h}B_{p}^{\alpha },$$

where *k*_h_ is the Steinmetz hysteresis coefficient and α is a numerical coefficient for a certain value of the peak magnetic flux density, *B_p_*.

The empirical parameters from (1) were determined for various types of materials, considering the saturation value of the magnetic flux density, the relative permeability, and other factors [29,30]. For example, the authors of [31] indicate that the coefficient α has a value of 1.64 for magnetization lower than 1 T and 2.60 for higher values, in the case of MnZn and FeSi alloys (here, it was considered that switching point of 1 T is the knee of the magnetization curve). In [31], different values of the temperature and pressure were considered for the Somaloy 3P 700 sample and the α is between 1.64 and 1.71. For these reasons, in this paper, α will be considered as 1.64.

The Steinmetz model has suffered many alternations over the years, but recently only two versions have been adopted. A first one considers that the total energy losses are divided in hysteresis losses (*W*_H_) and dynamic losses (*W*_D_) [32,33]:

(2) $$W_{\mathrm{tot}}=W_{H}+W_{D}=k_{h}\cdot B_{p}^{1.64}+k_{D}\cdot f\cdot B_{p}^{2},$$

where *k*_D_ is the Steinmetz coefficient for dynamic phenomena and *f* is the frequency of the signal.

More often, it is common to consider that the dynamic magnetic phenomena can be divided into eddy current losses (*W*_E_) and excess (abnormal) losses (*W*_A_) [33]:

(3) $$W_{\mathrm{tot}}=W_{H}+W_{E}+W_{A}=k_{h}\cdot B_{p}^{1.64}+k_{e}\cdot f\cdot B_{p}^{2}+k_{a}\cdot f^{0.5}\cdot B_{p}^{1.5},$$

where *k*_e_ is the Steinmetz eddy current coefficient and *k*_a_ is the excess losses coefficient.

**Figure 6:**
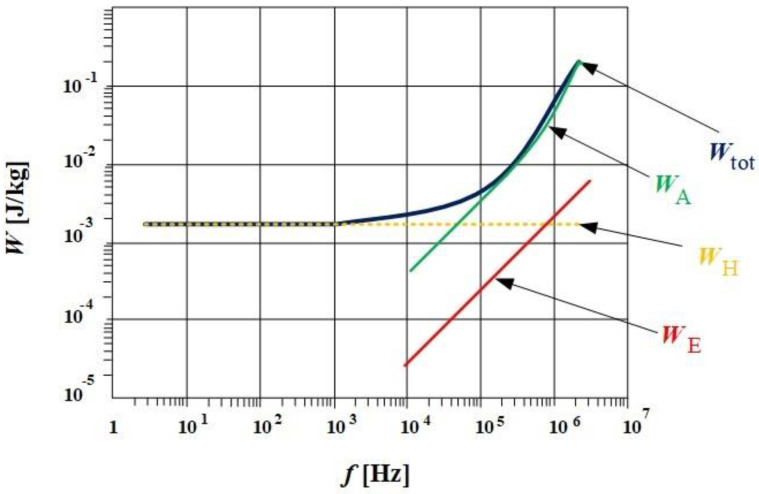
Exemplification of the procedure for separating energy losses for a certain value of the peak flux density.

Separation of the total energy losses in three terms indicated the three levels of the magnetization processes: geometry for the sample is associated with the classical energy losses (the macroscopic scale); the pinning of the domain walls in the lattice’s impurities is associated with the hysteresis energy losses (the microscopic scale); and the magnetic domains scale is directly connected with the excess energy losses (the mesoscopic scale) [1]. A graphical interpretation of the energy losses separation procedure can be followed in Figure 6 [32,33,34,35].

In conclusion, in the final form, in order to estimate the total energy losses, it is necessary to determine three coefficients (*k*_h_, *k*_e_, *k*_a_). The separation of losses presented above is also supported by Bertotti’s theory [36]. Here, too, measuring the area of the hysteresis loop at the lowest possible frequency (close to DC) will provide one with the hysteresis losses, *W*_H_. Then, by knowing some data about the sample (i.e., the thickness, *d*; the electrical conductivity, *σ*; and the mass density, *δ*) one can determine the losses due to the eddy currents, *W*_E_. Experimentally, it was found that the sum of these two terms do not represent the total energy losses. For this reason, abnormal and/or excess losses are introduced, *W*_A_. Considering these aspects, the total magnetic energy losses can be calculated as the following equation:

(4) $$W_{\mathrm{tot}}=W_{H}+W_{E}+W_{A}=W_{H}+\frac{\pi^{2}\cdot \sigma \cdot d^{2}\cdot B_{p}^{2}\cdot f}{6\cdot \delta }+8\sqrt{\sigma GSV_{0}}\cdot B_{p}^{1.5}f^{0.5},$$

where *S* is the section of the sample, *V*_0_ is an intrinsic parameter of the material depending on its microstructure, and *G* is a dimensionless parameter determined by Bertotti [36,37] with the value 0.1356.

## 4. Results and Discussion

Temperature is one important parameter that influences the magnetic behaviour of a material, simply by modifying its magnetic properties: a nonlinear evolution of magnetic parameters (saturation magnetization, relative permeability, coercive field) as function of temperature exists [38,39,40,41,42,43]. Heat dissipation is of paramount importance, as any change in both permeability and coercivity with temperature is reflected on magnetic losses. This influence on dynamic losses is related to eddy currents and wall motion effects. Eddy currents (also called Foucault’s currents) are loops of electrical current induced within conductors by a changing magnetic field in the conductor according to Faraday’s law of induction. The term eddy current comes from analogous currents seen in water in fluid dynamics, causing localised areas of turbulence known as eddies giving rise to persistent vortices. The current flowing through the resistance of the conductor also dissipates energy as heat in the material, and there is a compromise between the point of operation, size, frequency, and temperature. Therefore, the dynamic and static magnetic behaviour of materials is critical in order to decide their use in specific applications.

Losses in magnetic cores may be separated into three categories, with respect to the physical mechanism: static hysteretic losses, the classical eddy current losses, and the excess eddy current losses (or anomalous losses). The Bertotti–Fiorillo–Mazzetti model [44], in which the energy dissipation in materials under alternating magnetic field is due to eddy currents generated during motion of the magnetic domain, and the motion is non-sinusoidal, non-uniform, and non-repetitive, considers domain structure to be the main reason for the difference in excess eddy current losses. The magnetic domain structure was examined by magneto-optical Kerr microscope and it was found that the main domains are typically a few hundred of µm long and just a few µm wide, separated by 180° walls, while some parts of the FINEMET ribbon have a labyrinth-like domain structure. Such results are congruent with previous literature data [44,45]. It was previously shown that the domain structure would suffer certain transformations owing to the sample size, induction amplitude, as well as other conditions and/or parameters [45,46,47,48], leading to difficulties concerning total magnetic losses separation. To recall, power losses in soft magnetic materials are generated by hysteresis phenomenon and eddy currents, flowing in different scales as in the whole material volume, around moving domain walls or micro-currents related to the Barkhausen jumps. Some authors propose considering all types of power losses separately [48], while others suggest that these should be considered as a total [49]. Modelling of power losses is thus an interesting issue both for scientists and researchers, and there are many approaches to the modelling of power losses.

In this paper, we focus on the experimental results for FINEMET ribbon obtained with the previously described set-up (Figure 2). Four data sets were recorded for the magnetic flux densities (50 mT, 100 mT, 200 mT, and 300 mT, respectively) in a large frequency range (from 5 Hz to 900 kHz). Performing a fitting procedure using Matlab [5], we determine the three parameters in a first iteration. Table 1 presents these values.

**Figure 7:**
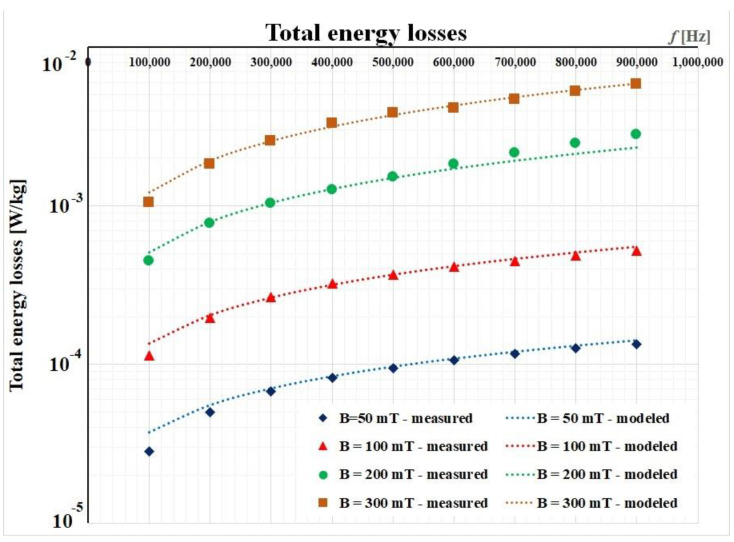
Initial fitting procedure for the FINEMET ribbon.

The graphical results after implementing this procedure are depicted in Figure 7. Here, we considered only the frequency range between 100 kHz and 900 kHz, because this represents the working range of planar transformers.

To evaluate the fitting accuracy, it is recommended to use the goodness-to-fit statistics. From the Curve Fitting Toolbox of Matlab, we have considered the following parameters:

– SSE = sum of squares due to error;
– R-square;
– RMSE = root mean squared error.

Table 2 presents the values of these fitting parameters for the initial iteration.

SSE represents the total deviation of the response values from the fit to the response values. A value closer to 0 indicates that the model has a smaller random error component [50]. In all four studied cases, this parameter is smaller than 10^−7^, so it is an indicator of a good fitting procedure. R-square is the square of the correlation between the response values and their predicted ones [50]. An R-square value over 0.98 means that the fit explains 98% of the total variation in the data about the average; this coefficient also indicates a good fitting. RMSE is an estimate of the standard deviation of the random component in the data [50]. An RMSE value closer to 0 indicates a fit that is more useful for prediction.

The next step was to identify a function for each parameter in order to estimate the total energy losses for higher values of the magnetic flux density. Owing to the lack of measured data (only four values for each parameter), we have chosen a linear variation for each parameter:

(5) $$k_{h}\left(B\right)=0.001343\times B+0.000111$$

**Figure 8:**
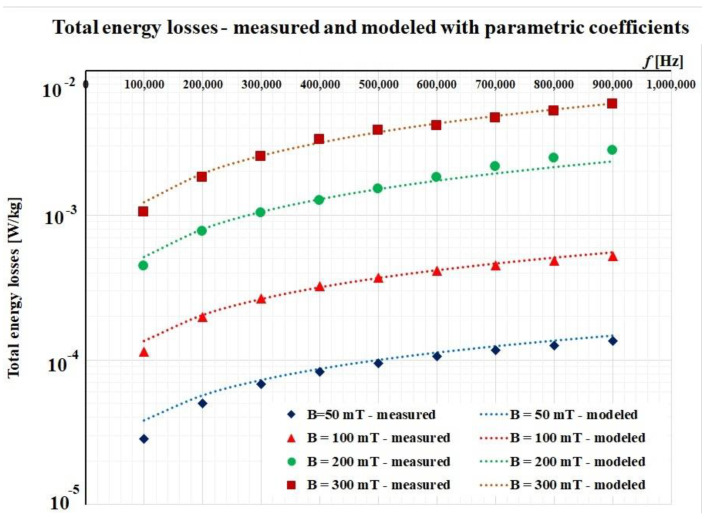
Fitting procedure for the FINEMET ribbon using parametric coefficients.

After this iteration, the results are also good and they are presented in Figure 8.

**Figure 9:**
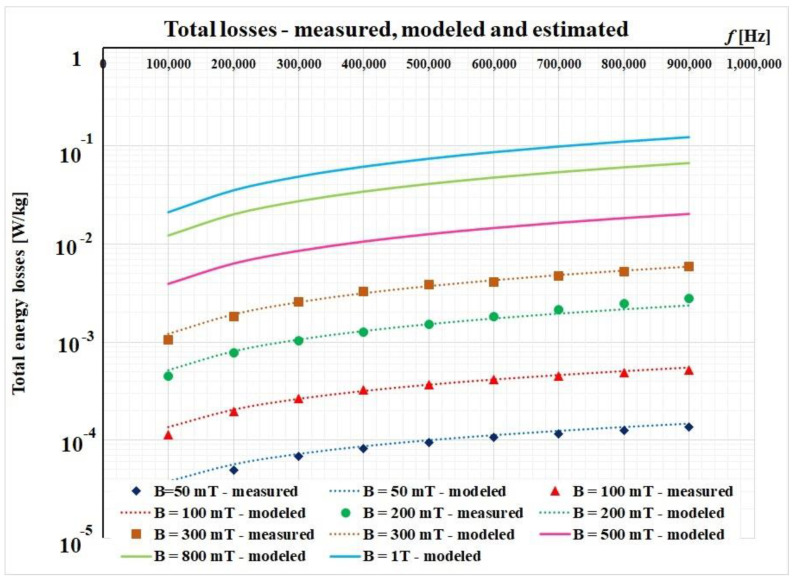
Estimation of total energy losses for 500 mT, 800 mT, and 1 T, respectively.

By using such linear approximations, we were able to perform an estimation of the total magnetic losses that occur at higher values of the magnetic flux densities. Figure 9 represents the first four curves (measured and modelled) and a family of three other curves for 500 mT, 800 mT, and 1 T, respectively.

**Figure 10:**
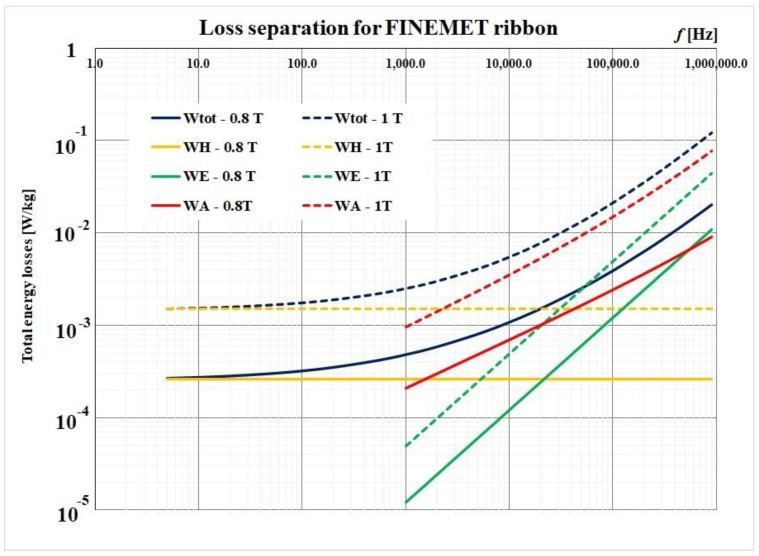
Loss separation procedure for 500 mT and 1 T, respectively.

Using Equation (4), we performed a losses separation procedure for 800 mT and 1 T, respectively. The following data for the investigated nanocrystalline ribbon were considered: mass density *δ* = 7730 kg/m^3^, electrical conductivity *σ* = 141 µΩcm, and the ribbon thickness *d* = 18 µm. The results are presented in Figure 10.

The total magnetic losses separation is a procedure that offers us important information about the contribution of each type of magnetic losses in a material. By extrapolation to the frequency of 0 Hz, the hysteresis losses are determined. The eddy current losses can also be calculated with the ration from the second term of the relation (4). What remains of the total losses represents the excess losses. These become important with increasing frequency (over 10 kHz) and they highlight the movement of the interdomenial walls of the magnetic material. These losses depend significantly on the size and arrangement of the magnetic fields. The high values of these losses for frequencies of the order of the tens and hundreds of kHz indicate fairly large magnetic domains compared with the thickness of a magnetic layer. Therefore, for such materials, these losses cannot be ignored.

Over a wide frequency range, the coefficient of inductance (i.e., µ_r_) remarkably decreases as the stress is increased in the samples (data not shown here). The reason for the observation of lower permeability in stress-annealed samples is that a transverse magnetic anisotropy is induced in the nanocrystalline alloy after stress annealing and, consequently, it becomes more difficult to magnetize the alloy in the direction of the magnetization field, thus the magnetic permeability is diminished. However, the results are beyond the interest of this study and will be further and thoroughly discussed elsewhere. To conclude in brief, FINEMET-type alloys were measured and modelled, and the magnetic properties of this material were further discussed using the Steinmetz procedure. This approach is also suitable for thin films, such as in pulsed laser deposited films and multi-layered assemblies, where accurate control of the nanocrystalline structuring may be achieved.

## 5. Conclusions

Determining the characteristics of soft materials that are used in electromagnetic devices is a present issue. The characterization of the magnetic properties within the limits offered by the experimental devices, followed by their modelling and estimation, is the subject of this paper. The studied material is a FINEMET-type alloy that could be characterized for magnetic inductions up to 300 mT and frequencies of 900 kHz. However, in devices such as planar transformers, the core is subjected to magnetic flux densities values of about 500 mT–1 T (considering that the saturation value for these materials is near 1.2 T). An estimation of the magnetic energy losses using the Steinmetz procedure was used for these values. The specific coefficients were adjusted using an iterative fitting procedure. The results obtained for the values of the magnetic flux densities, where experimental determinations were performed, offered very low relative errors in the range 100 kHz–900 kHz. This aspect was highlighted by the analysis of some statistical parameters for evaluating the goodness of the fitting procedure. This approach is also suitable for thin films, such as in pulsed laser deposited films and multi-layered assemblies, where accurate control of the nanocrystalline structuring may be achieved. Finally, a total loss separation procedure was performed for two of the estimated values. These findings suggest that the application of this procedure could be suitable for cases when the experimental set’s limits must be over-fulfilled.

## Figures and Tables

**Table 1:** Steinmetz coefficients after initial iteration.

B [mT]	*k*_h_ [-]	*k*_e_ [-]	*k*_a_ [-]
50	1.67 × 10^−4^	2.18 × 10^−8^	8.65 × 10^−6^
100	2.54 × 10^−4^	2.67 × 10^−8^	1.02 × 10^−8^
200	3.90 × 10^−4^	3.54 × 10^−8^	1.20 × 10^−8^
300	5.06 × 10^−4^	4.39 × 10^−8^	1.46 × 10^−8^

**Table 2:** Parameters used for identifying the goodness of the fitting.

B [mT]	SSE	R	RMSE
50	2.75 × 10^−10^	0.9945	3.59 × 10^−6^
100	3.17 × 10^−9^	0.9957	1.23 × 10^−5^
200	2.76 × 10^−7^	0.9842	1.12 × 10^−4^
300	2.26 × 10^−7^	0.9973	1.04 × 10^−4^

## Data Availability

Not applicable.
